# The American Medical Profession

**Published:** 1882-04

**Authors:** 


### Editorial.

### THE AMERICAN MEDICAL PROFESSION.

At the recent commencement exercises we listened to an eulogy upon the medical profession of this country, in which we, as a profession, received favorable comparative criticism. Our professional standing, as compared with that of our brothers across the water, may be considered as uncertain, to say the least. It is our present purpose to present a single bearing of this many-sided question. However wide our views upon the question as a whole may differ, there can not possibly be any dissent from the statement that in original research into the sciences of life and disease, such as biology and pathology, including of course many sub-departments, we bear only a very unfavorable comparison.

We believe, and that it can truthfully be said, that there is not a university town in Europe that does not furnish a larger number of medical men, engaged exclusively or very nearly so in the labor of original research, than the whole of the United States. Where are our physiological and pathological laboratories that would not appear preposterously insignificant in comparison with those of such men as Stricker, Donders and Rindfleisch.

The fact cannot be denied. We proceed, therefore, to find the reason. The common excuse that our country is still too new and undeveloped, the population too primitive and scattered, is no longer available. The fault lies with the profession itself. A recent meeting of the Medical Association of this city will serve to illustrate. A certain gentleman of this city was announced as having consented to read a paper, the subject of his paper being the relation of the various so-called germs to the etiology of disease. Certainly no subject could have been more interesting and important, the essayist a gentleman than whom there is no one in this city better qualified for the task of presenting the subject in an authoritative and exhaustive manner. Considering these facts, is it not a remarkable and humiliating thought that at the time first appointed no quorum was present, and at the adjourned meeting there were present less than a dozen all told. Could any subject have been more attractive? Is there any regarding which there exists greater ignorance, both among the laity and the profession? If it be true that the greater the ignorance the greater the assurance, then we find here an apt illustration, there being no subject regarding which more reckless assertions and foolish statements are daily made by press and people.

The essayist's failure to attract from the ranks of a numerous profession a larger audience is no exception, but on the contrary the unvarying rule shows but too clearly the almost hopeless apathy touching scientific matters which pervades the medical profession of this country. It may be claimed that we are not ignorant of the results obtained in the various European laboratories, and that we are not slow to profit and make practical application of any facts determined or discoveries made. But this will not excuse our idleness. The profession of other countries has a right to look to us for a return of original information, and our duty to humanity and our zeal for the progress of medicine should further incite us.

As to causes which have been active in bringing about this condition of inactivity and stagnation as well as the only remedy which in our opinion can be effectual, we have clearly defined views, the presentation of which we reserve for a future editorial.

